# Danish mothers of young children adhere less to international physical activity guidelines compared with mothers of older children

**DOI:** 10.1016/j.pmedr.2025.102970

**Published:** 2025-01-11

**Authors:** Solvej Videbæk Bueno, Sebastian Deisting Skejø, Rasmus Oestergaard Nielsen, Knud Ryom, Per Kallestrup, Peter Elsborg, Christina Bjørk Petersen, Julie Sandell Jacobsen

**Affiliations:** a Department of Public Health, Aarhus University, Bartholins Allé 2, 8000 Aarhus C, Denmark; b Research Unit for General Practice, Bartholins Allé 2, 8000 Aarhus C, Denmark; c Center for Clinical Research and Prevention, Copenhagen University Hospital – Bispebjerg and Frederiksberg, Capital Region of Denmark, Copenhagen, Denmark; d National Institute of Public Health, Faculty of Health Science, University of Southern Denmark, Copenhagen, Denmark; e Research Centre for Activity and Prevention, Faculty of Health and Society, VIA University College, Aarhus, Denmark

**Keywords:** Women's health, Exercise, Mothers, Parity, Infant, Health priorities

## Abstract

**Introduction:**

This study aimed to investigate if the prevalence proportion of non-adherence to the World Health Organization (WHO) guidelines of a minimum of 150 minutes of moderate-to-vigorous physical activity per week varied among mothers based on the age of their youngest child. Additionally, the study aimed to investigate whether such association varied by parity.

**Methods:**

The population-based design used self-reported physical activity data on 8774 Danish mothers who participated in the Danish National Health Survey 2021. The primary outcome was weekly hours of moderate-to-vigorous physical activity, dichotomized into adherers or non-adherers to WHO guidelines.

**Results:**

The prevalence proportions of non-adherence ranged from 60 % to 70 %. Significantly higher prevalence proportions of non-adherers were observed among mothers of infants and toddlers aged 0–3 years compared with mothers of school-age children. When stratified by parity, multipara mothers with infants aged 0–6 months reported the highest prevalence proportion (74 %) of non-adherence among all subgroups.

**Conclusion:**

Due to the health benefits derived from adequate levels of physical activity, the large proportion of mothers not adhering to the WHO physical activity guidelines is a public health concern. The findings of the present study suggest a need for improving interventions and policies to enhance physical activity levels in mothers, especially mothers of infants and toddlers.

## Highlights

• The majority of mothers do not meet international physical activity guidelines
• Mothers of infants and toddlers show significantly higher non-adherence proportions
• Multipara mothers with infants were the most non-adherent sub-group (74 %)
• Interventions to boost physical activity in mothers of young children are needed

## 1 Introduction

Physical activity impact human health and well-being (Bull et al., 2020). The World Health Organization (WHO) recommend that adults aged 18–64 years engage in a weekly minimum of 150–300 minutes of moderate-intensity aerobic physical activity (MPA), 75–150 minutes of vigorous-intensity aerobic physical activity (VPA), or an equivalent combination of moderate- and vigorous-intensity aerobic physical activity (MVPA) (World Health Organization, 2020). However, substantial proportions of the population do not adhere to the recommended physical activity level, and large gender disparities exist (Guthold et al., 2018). Across the globe, including in Denmark, women adhere less to the WHO physical activity guidelines compared with men (World Health Organization, 2020; Guthold et al., 2018). This gender disparity may be related to cultural norms, traditional roles, or lack of social and community support (Guthold et al., 2018), but also the life circumstances of women, such as pregnancies, childbirths, and childcare responsibilities may play an important role in this disparity (Abbasi and Van Den Akker, 2015; Makama et al., 2021; Brown and Trost, 2003). In 2020, specific recommendations on physical activity for pregnant and postpartum women were for the first time provided by the (World Health Organization, 2020). Accordingly, pregnant and postpartum women are recommended to engage in at least 150 minutes of MPA per week. Later in parenthood, women are recommended to follow the physical activity guidelines for all adults to achieve the established health-related benefits from regular physical activity (Bull et al., 2020).

Previous literature reviews have reported that considerable proportions of mothers do not adhere to the WHO physical activity guidelines (Bellows-Riecken and Rhodes, 2008; Juwono et al., 2021). Similar, a recent population-based study in Denmark, involving 20,022 women aged 20–40 years, revealed that mothers face a 24 % higher risk of non-adherence to the WHO physical activity guidelines than their nulliparous peers (Bueno et al., 2024). Mothers represent a considerable population subgroup, and non-adherence to the international physical activity guidelines is a public health concern. In Denmark, with a population of about six million people (Statistics Denmark, 2024), approximately 60,000 women give birth annually (Statistics Denmark, 2023). Consequently, mothers constitute more than one million individuals, and approximately 180,000 of these have young children below three years of age.

Notably, research has shown that physical activity in mothers is linked to enhanced emotional well-being (Blum et al., 2004; Currie, 2004), decreased risk of postpartum depression (Kołomańska-Bogucka and Mazur-Bialy, 2019; Pivarnik et al., 2006; Norman et al., 2010), and lower risk of gestational weight gain retention (van der Pligt et al., 2013). Additionally, physical activity not only enhances the overall well-being of mothers but is also positively associated with the health of their children (Gustafson and Rhodes, 2006; Petersen et al., 2020). Population-based research conducted in the United Kingdom (Hesketh et al., 2019) and Switzerland (Bringolf-Isler et al., 2018) has demonstrated that early-school-aged children of physically active mothers tend to exhibit higher levels of physical activity themselves.

To effectively target interventions to increase physical activity among mothers, it is imperative to investigate whether certain subgroups of mothers require such interventions more urgently than others. Research from Canada (Adamo et al., 2012; Candelaria et al., 2012; Gaston et al., 2014), Australia (Makama et al., 2023), the USA (Kwon et al., 2023), and the United Kingdom (Simpson et al., 2022) suggests that higher risk of non-adherence to physical activity guidelines exists among mothers of young children compared with mothers of older children. Given the international variations in maternity care programmes, maternity leave, and childcare (Waldfogel, 2001), large-scale population-based studies are needed to explore the association between maternal adherence to WHO physical activity guidelines and the age of the youngest child in the Nordic countries. To our knowledge, only one Nordic study has been conducted; a Finnish longitudinal cohort study (Palomäki et al., 2023) based on data from 400 women reporting their physical activity engagement in 1992, 2001, 2007, and 2011. This study found that mothers of young children tended to be less likely to meet the recommended physical activity level. Additional research within Nordic populations would bolster the rationale for developing local strategies aimed at promoting physical activity among specific sub-groups of mothers.

The present study aimed to investigate if the prevalence proportion of Danish mothers not adhering to WHO physical activity guidelines varies based on the age of their youngest child, including infants aged 0–6 months, infants aged 6–12 months, toddlers aged 1–3 years, pre-school children aged 3–6 years, and schoolchildren aged 6–13 years. Additionally, the study aimed to investigate if such association among Danish mothers varies by parity.

## 2 Methods

### 2.1 Study population

This population-based cross-sectional study is based on data from the Danish National Health Survey (DNHS) 2021 (Christensen et al., 2022). The DNHS is conducted every four years in a representative sample of the adult Danish population (age 16 years and above) extracted from the Danish Civil Registration System (Pedersen, 2011). The design of the DNHS has been described elsewhere (Christensen et al., 2020; Rosendahl Jensen, 2022).

The population of interest was Danish women aged 16–40 years who gave birth to at least one live singleton child and completed the DNHS in 2021. The childbirth status of each woman was identified by linkage to the Danish Medical Birth Register (MBR) (Bliddal et al., 2018) through the civil personal registration (CPR) number, which is a unique 10-digit personal identifier code assigned to all Danish citizens (Pedersen, 2011). The MBR holds medical and civil information on all births in Denmark and is maintained by the Danish Health Data Authority (Bliddal et al., 2018).

### 2.2 Ethical approval

Ethical approval for the study was provided by the legal department at the University of Southern Denmark. The study is registered in the Record of Processing Activities at the Research Unit of General Practice in Aarhus in accordance with the provisions of the General Data Protection Regulation (GDPR). Moreover, according to Danish law, individual-level linkage of data from, for example, surveys and administrative registries is allowed without further consent when it is for research purposes and when thoroughly ensuring that results are presented in an anonymized way. The study adhered strictly to these legal and ethical guidelines, ensuring the privacy and confidentiality of all participants.

### 2.3 Outcome: physical activity

The number of weekly hours of self-reported leisure-time MVPA and VPA was based on the following questions in the DNHS 2021: “In a typical week, how much time do you spend on moderate and vigorous physical activity in which you become short of breath?” and “How much of the time, stated in the previous question, do you spend on vigorous physical activity, in which you become so short of breath that it is difficult to speak?” For MVPA, the response options were categorized into five categories: (1) less than 30 minutes, (2) 30–89 minutes, (3) 90–149 minutes, (4) 150–299 minutes, and (5) 300 minutes or more. For VPA, the response options were: (1) less than 30 minutes, (2) 30–59 minutes, (3) 60–89 minutes, (4) 90–149 minutes, and (5) 150 minutes or more. The validity of the questions assessing physical activity has been reported in previous papers (Danquah et al., 2018). Based on the WHO physical activity guidelines of a minimum of 150-300 minutes of MPA, 75-150 minutes of VPA, or an equivalent combination of MVPA per week, the weekly hours of self-reported leisure-time physical activity were dichotomized into: (1) mothers not adhering to the WHO physical activity guidelines and (2) mothers adhering to the WHO physical activity guidelines. The classification of adherence or non-adherence to the WHO physical activity guidelines based on the questions is described in detail in a previous paper (Bueno et al., 2024).

#### 2.3.1 Exposure: age of the youngest child

The primary exposure variable was the age of the youngest child, which was obtained from the MBR (Bliddal et al., 2018). The systematic nature of the MBR provided a highly valid method for obtaining the age of the children (Schmidt et al., 2019). Mothers were categorized, based on the age of their youngest child, into five groups: mothers of infants aged 0–6 months, mothers of infants aged 6–12 months, mothers of toddlers aged 1–3 years, mothers of pre-school children aged 3–6 years, and mothers of school children aged 7–13 years. This categorization is based on the typical age of children attending nursery, kindergarten, and school in Denmark (Lifeindenmark, 2024; Denmark, 2024b). Moreover, infants (0–1 year) were divided into two groups, 0–6 months and 6–12 months, due to an assumed considerable change in infant sleeping and eating patterns at this age, which could influence the mother's ability to engage in leisure-time physical activities. Due to the size of the dataset and restrictions in the General Data Protection Regulation (Union, 2016), we were not able to include mothers of children aged 14–18 years.

#### 2.3.2 Exposure: parity

Parity was extracted from the MBR (Bliddal et al., 2018), and the mothers were categorized into two groups based on their number of children: first-time mothers and multipara, the latter representing mothers of two or more children.

### 2.4 Demographics and maternal characteristics

To describe the study population, the following demographic covariates were extracted from Danish administrative registries through the CPR number (Pedersen, 2011): age, cohabitation, educational level, country of origin, urbanization, household income, and employment status.

### 2.5 Statistical analyses

A priori, we had decided to divide child age into categories if the relation between child age and the prevalence proportion of maternal non-adherence to WHO guidelines appeared to be non-linear upon visual inspection. Consequently, we used binomial regression with the log-link function and the age of the youngest child as the independent variable to estimate the prevalence proportion ratios of maternal non-adherence to WHO guidelines according to the age of the youngest child, with age 6–13 years as the reference group. We computed the prevalence proportion for each age category as the estimated marginal means using the emmeans package (Carroll et al., 2011). We computed the prevalence proportion differences using the riskdiff function of the risks package (Rasmussen et al., 2024). To provide context for the analyses, we added the proportion of non-adherence to WHO physical activity for nulliparous women based on findings from a previous published paper using data from the DNHS 2021 (Bueno et al., 2024).

To investigate differences between first-time mothers and multipara, we performed the same analysis stratified by parity in the regression model. The estimated coefficient represents the between-parity ratio of the between-age prevalence proportion ratios for the given child age. We computed the within-strata prevalence proportion ratio (i.e. the prevalence proportion of a given child age relative to the prevalence proportion of the same stratum in the reference group) as the contrast of the estimated marginal means conditional on parity by using the emmeans package in R (Lenth, 2024). We performed five similar additional analyses based on cohabitation (living alone, living with someone), educational level (≤10 years, 11–15 years, ≥16, and unknown), country of origin based on registry data from Statistics Denmark using the parent's nationality or country of birth for the grouping (Denmark, other Western, non-Western), residence urbanization (≥100,000, 20,000-100,000, 1000-20,000, <1000 inhabitants), and family income (1st, 2nd, and 3rd tertile), respectively (Supplementary Table A-E).

All statistical analyses were performed using R Statistical Software (version 4.4.1, https://www.r-project.org/), and the alpha level for statistical significance was set at 0.05. The analysis code is available from https://osf.io/mq5p8/.

## 3 Results

### 3.1 Sample characteristics

**Fig. 1:**
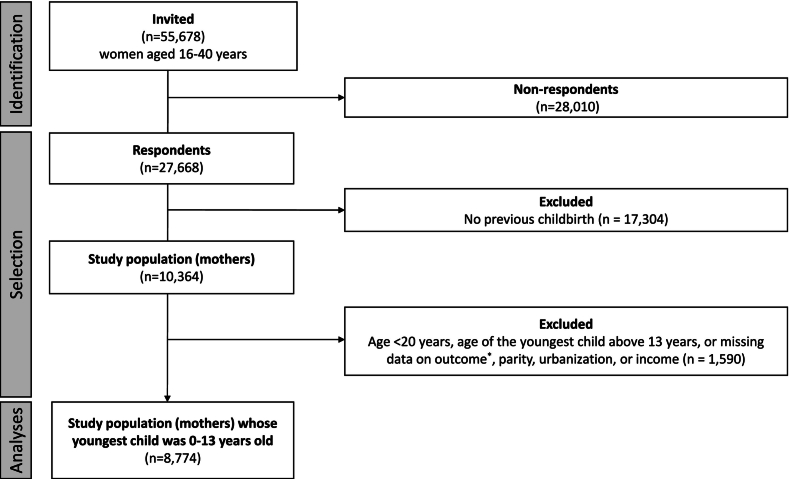
Flowchart of sampling process for mothers participating in the Danish National Health Survey 2021. *Non-adherence to the World Health Organization physical activity guideline of a weekly minimum of 150 minutes of moderate-to-vigorous physical activity.

In total, 55,678 women aged 16–40 years were invited to complete the DNHS 2021 (Rosendahl Jensen, 2022). Of these, 45,314 were excluded, 28,010 did not respond to the DNHS questionnaire on physical activity in 2021, and 17,304 had no previous childbirth. Consequently, 10,364 women were eligible for inclusion (Fig. 1). A further 1590 women were excluded due to age > 13 years of the youngest child, missing data on adherence to the WHO physical activity guidelines, parity or income, and GDPR restrictions due to small number of individuals (i.e. mothers aged <20, or unknown urbanization). Finally, 8774 mothers were included in the main analyses.

Descriptive characteristics of the study population are presented in Table 1. Among the study participants, 28 % were mothers having a school-aged child as their youngest. Of the mothers not adhering to WHO physical activity guidelines, 35 % were first-time mothers, while 65 % were multipara. The general representativeness of the respondents in the DNHS 2021 has been described in a previous publication (Jezek et al., 2024).

**Table 1:** Characteristics of mothers participating in the Danish National Health Survey 2021.

N (%)						
	Age of youngest child 0–6 months 675 (7.7)	Age of youngest child 6–12 months 778 (8.9)	Age of youngest child 1–3 years 2540 (28.9)	Age of youngest child 3–6 years 2325 (26.5)	Age of youngest child 6–13 years 2456 (28.0)	Total 8774 (100)
**Parity** First-time mothers Multipara	321 (47.6) 354 (52.4)	359 (46.1) 419 (53.9)	1049 (41.3) 1491 (58.7)	657 (28.3) 1668 (71.7)	665 (27.1) 1791 (73.2)	3051 (34.8) 5723 (65.2)
**Age group** (years) 20–25 26–30 31–35 36–40	64 (9.5) 257 (38.1) 235 (34.8) 119 (17.6)	46 (5.9) 256 (32.9) 325 (41.8) 151 (19.4)	126 (5.0) 715 (28.1) 1099 (43.3) 600 (23.6)	34 (1.5) 281 (12.1) 896 (38.5) 1114 (47.9)	10 (0.4) 79 (3.2) 550 (22.4) 1817 (74.0)	280 (3.2) 1588 (18.1) 3105(35.4) 3801 (43.3)
**Cohabitation**^a^ Living alone Living with someone	32 (4.7) 643 (95.3)	29 (3.7) 479 (96.3)	155 (6.1) 2385 (93.9)	320 (13.8) 2005 (86.2)	550 (22.4) 1906 (77.6)	1086 (12.4) 7688 (87.6)
**Educational level** (years) ≤10 11–15 ≥16 Unknown	60 (8.9) 226 (33.5) 331 (49.0) 58 (8.6)	44 (5.7) 233 (29.9) 426 (54.8) 75 (9.6)	165 (6.5) 834 (32.8) 1362 (53.6) 179 (7.1)	196 (8.4) 837 (36.0) 1160 (49.9) 132 (5.7)	246 (10.0) 1071 (43.6) 1039 (42.3) 100 (4.1)	711 (8.1) 3201 (36.5) 4318 (49.2) 544 (6.2)
**Country of origin** Denmark Other Western Non-Western	572 (84.8) 40 (5.9) 63 (9.3)	667 (85.7) 40 (5.2) 71 (9,1)	2192 (86.3) 144 (5.7) 204 (8.0)	1981 (85.2) 122 (5.3) 222 (9,5)	2185 (89.0) 88 (3.6) 183 (7.4)	7597 (86.6) 434 (4.9) 743 (8.5)
**Urbanization** ≥ 100,000 20,000–100,000 1000–20,000 <1000	240 (35.5) 116 (17.2) 193 (28.6) 126 (18.7)	265 (34.1) 125 (16.1) 232 (29.8) 156 (20.0)	824 (32.4) 452 (17.8) 794 (31.2) 470 (18.5)	634 (27.3) 441 (19.0) 799 (34.3) 451 (19.4)	568 (23.1) 429 (17.5) 932 (37.9) 527 (21.5)	2531 (28.9) 1563 (17.8) 2950 (33.6) 1730 (19.7)
**Income** (tertiles) 1st 2nd 3rd	240 (35.5) 198 (29.4) 237 (35.1)	240 (30.9) 268 (34.4) 270 (34.7)	798 (31.4) 889 (35.0) 853 (33.6)	746 (32.1) 771 (33.2) 808 (34.7)	842 (34.3) 804 (32.7) 810 (33.0)	2866 (32.7) 2930 (33.4) 2978 (33.9)
**Employment status** Employed Unemployed^b^	517 (76.6) 158 (23.4)	601 (77.2) 177 (22.8)	1962 (77.2) 578 (22.8)	1916 (82.4) 409 (17.6)	2008 (81.8) 448 (18.2)	7004 (79.8) 1770 (20.2)

a Based on civil status and family type.

b Including women enrolled in education and social security.

### 3.2 Analyses

**Fig. 2:**
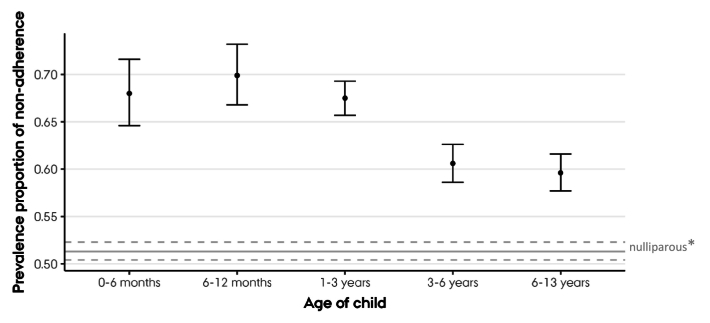
Proportions and 95 % confidence intervals of Danish mothers not adhering to the World Health Organization guidelines of a weekly minimum of 150 min of moderate-to-vigorous physical activity across the five youngest child age groups. * The proportion and 95 % confidence intervals of Danish nulliparous women not adhering to the World Health Organization guidelines of a weekly minimum of 150 min of moderate-to-vigorous physical activity (results published in a previous paper)^a^. a. Bueno SV, Nielsen RO, Kallestrup P, et al. Parous women perform less moderate to vigorous physical activity than their nulliparous peers: a population-based study in Denmark. *Public Health* 2024; **231**: 47–54.

Table 2 presents the proportions of mothers not adhering to WHO physical activity guidelines across the five youngest child age groups. The prevalence proportions of non-adherence ranged from 60 % to 70 %. The highest prevalence proportion (70 %) was observed in mothers of children aged 6–12 months, whereas the lowest (60 %) was seen in mothers with children aged 6–13 years (reference group), corresponding to a 17 % higher risk of non-adherence to the WHO physical activity guidelines among mothers of the youngest children compared with mothers of school-aged children. Fig. 2 shows that each subgroup of mothers having children in the three youngest age groups stand out by all having a significantly higher risk of non-adherence to the WHO physical activity guidelines compared with the reference group. Nonetheless, the overall proportion of non-adherence among mothers is higher than that observed among nulliparous women, as visualized (Fig. 2).

**Table 2:** Association between non-adherence to the World Health Organization physical activity guidelines* and age of youngest child in mothers participating in the Danish National Health Survey 2021.

	Non-adherers/total	Prevalence Proportion % (95 % CI)		Prevalence Proportion Ratio (95 % CI)		Prevalence Proportion Difference % (95 % CI)	
Age of youngest child							
0–6 months	459/675	68.0	(64.6;71.6)	1.14	(1.07;1.1.21)	8.4	(4.4;12.4)
6–12 months	544/778	69.9	(66.8;73.2)	1.17	(1.11;1.24)	10.3	(6.6;14.1)
1–3 years	1715/2540	67.5	(65.7;69.3)	1.13	(1.09;1.18)	7.9	(5.2;10.5)
3–6 years	1409/2.325	60.6	(58.6;62.6)	1.02	(0.97;1.07)	1.0	(−1.8;3.8)
6–13 years	1464/2456	59.6	(57.7;61.6)	1.00		0.0	

⁎ The World Health Organization recommend a weekly minimum of 150 minutes of moderate-to-vigorous physical activity.

Table 3 presents the prevalence proportion of mothers not adhering to the WHO physical activity guidelines across the five youngest child age groups, stratified by parity. This analysis shows that the relationship between child age and non-adherence was statistically significantly more pronounced for multipara than for first-time mothers of children aged 0–6 months, while there were similar, but smaller, non-significant differences for mothers of children aged 6–12 months and 1–3 years.

**Table 3:** Differences between non-adherence to the World Health Organization physical activity guidelines* and age of youngest child stratified by parity in mothers participating in the Danish National Health Survey 2021.

	Stratum 1 (first-time mothers)					Stratum 2 (multipara)					Ratio between strata of parity
	Non-adherers/ total	Prevalence Proportion % (95 % CI)		Prevalence Proportion Ratio (95 % CI)		Non-adherers/ total	Prevalence Proportion % (95 % CI)		Prevalence Proportion Ratio (95 % CI)		Ratio of Prevalence Proportion Ratio (Reference level: first-time mothers)
Age of youngest child											
0–6 months	198/321	61.7 (56.6;67.2)		0.98 (83.5,1.1)		261/354	73.7 (69.3;78.5)		1.27 (1.13;1.41)		1.30 (1.14;1.47)
6–12 months	248/359	69.1 (64.5; 74.0)		1.10 (0.96;1.26)		296/419	70.6 (66.4;75.1)		1.21 (1.09;1.35)		1.11 (0.99;1.24)
1–3 years	719/1049	68.5 (65.8;71.4)		1.09 (0.98;1.21)		994/1491	66.7 (64.4;69.2)		1.15 (1.06;1.24)		1.06 (0.97;1.15)
3–6 years	436/657	66.4 (62.8;70.1)		1.05 (0.93;1.19)		972/1668	58.3 (56.0;60.7)		1.00 (0.92;1.09)		0.95 (0.86;1.05)
6–13 years	420/665	63.2 (59.6;66.9)		1.00		1044/1791	58.3 (56.1;60.6)		1.00		–

⁎ The World Health Organization recommend a weekly minimum of 150 minutes of moderate-to-vigorous physical activity.

Several additional explorative supplementary analyses were completed to investigate whether the association between non-adherence to WHO physical activity guidelines and the age of the youngest child differed according to cohabitation (Supplementary Table A), educational level (Supplementary Table B), country of origin (Supplementary Table C), urbanization (Supplementary Table D) and family income (Supplementary Table E). These analyses are beyond the scope of this paper but are published as supplementary material as an attempt to add additional information from the dataset, which could be relevant to other researchers.

## 4 Discussion

### 4.1 Key findings

The present study showed that Danish mothers of infants and toddlers were significantly less likely to adhere to the WHO physical activity guidelines compared with mothers of older children. However, even among the most adherent group (mothers of school-age children), the prevalence proportion of non-adherers remained high at 60 %, raising concerns from a public health perspective. When stratified by parity, multipara mothers of infants aged 0–6 months reported the highest prevalence proportion of non-adherers (74 %) of all subgroups. This contrasts the finding that first-time mothers of children in the same age group reported the third-lowest prevalence proportion of non-adherers (62 %) of all subgroups.

### 4.2 Comparison with existing findings

Previous international studies, predominantly conducted in Australia, the USA, and Canada, have indicated a tendency that mothers of young children face increased risk of not adhering to the recommended level of physical activity (Adamo et al., 2012; Candelaria et al., 2012; Gaston et al., 2014; Makama et al., 2023; Kwon et al., 2023). For instance, a large Australian population-based cross-sectional study (Makama et al., 2023), which was based on self-reported physical activity data from 4290 women, reported that the proportion of non-adherers to the physical activity recommendations was 65 % in mothers of infants aged 0–6 months, 56 % in mothers of infants aged 6–12 months, 60 % in mothers of toddlers aged 1–3 years, and 54 % in mothers of pre-school children aged 3–6 years.

Similarly, two European population-based studies investigated the association between physical activity levels among mothers and the age of their children (Simpson et al., 2022; Palomäki et al., 2023). A Finnish longitudinal cohort study (Palomäki et al., 2023), involving 400 women, found that the probability of meeting the recommended physical activity level was 32 % for mothers of children aged 0–2 years, 46 % for mothers of children aged 3–5 years, and 52 % for mothers of children aged 6–17 years. In line with the findings from the present study, they revealed an elevated risk of non-adherence to the recommended physical activity level among mothers of infants and toddlers. However, the data in the Finnish study was collected in a period dating back to 1992, and societal changes have likely influenced parenthood, childcare, and adult physical activity patterns in the population over time. Interestingly, the Finnish study also reported the paternal adherence to the recommended physical activity level; this ranged from 47 % for fathers of children aged 0–2 years to 60 % for fathers of children aged 6–17 years. These results suggest that the adherence to the recommended physical activity level varied more among mothers than among fathers.

### 4.3 Clinical implications

Mothers of infants and toddlers exhibited the highest proportion of non-adherence to the WHO physical activity guidelines, possibly due to physiological changes, emotional alterations, and caregiving responsibilities during early motherhood (Makama et al., 2021; McIntyre and Rhodes, 2009; Mailey et al., 2014). Notably, the tendency among mothers to prioritize the health of their children and the well-being of the family above themselves has previously been described and termed ‘the burden of care’ (O'Brien et al., 2014). As children grow older, mothers may find opportunities to engage in higher-intensity physical activity through shared activities with their children or by allocating time for leisure-time physical activity outside the home. The cross-sectional design of the present population-based study limits our ability to infer causal relationships regarding the observed distribution of non-adherence to physical activity. Consequently, this study focuses on identifying subgroups of mothers who may be particularly relevant as target groups for future interventions aiming at increasing physical activity. Future research may explore the underlying causal mechanisms, including structural factors such as policies, community programmes or other societal influences, to better understand and address the differences in non-adherence to physical activity in subgroups of mothers.

In Denmark, a comprehensive maternity care programme is offered to all women giving birth, and new mothers are in close contact with healthcare providers in this period of life. Although, a recent study demonstrates that multiple gaps exist in the current available physical activity guideline materials for women in this specific subgroup, which makes it challenging for women and clinicians to navigate a safe and successful return to physical activity (Schulz et al., 2023). Furthermore, to provide optimal physical activity support for mothers of young children, the collaborative approach of co-creation may prove advantageous in identifying the most effective strategies (Leask et al., 2019).

We recently conducted a register study, involving 20,022 Danish women, and found that mothers engage less in MVPA, but more in light physical activity compared with their nulliparous peers (Bueno et al., 2024). Hence, future interventions aiming at increasing physical activity among mothers may benefit from increasing the physical activity intensity from light to moderate or vigorous to promote the adherence to recommended physical activity level and to gain the associated health benefits. Specifically, mothers of infants and toddlers emerge as a subgroup in this population that may require attention in physical activity-promoting interventions. To encourage mothers to transition from light physical activity to MVPA, effective strategies promoting activities that are practical and easily incorporated into daily routines are essential. These strategies should involve collaboration with healthcare users, respected community leaders, and well-trained staff who are representative of the target population. Therefore, we recommend that researchers, health professionals, healthcare users, and local communities work together to develop MVPA interventions targeted mothers, and especially mothers of infants and toddlers (Carroll et al., 2011; Rasmussen et al., 2024).Given that caregiving dynamics has changed in some countries, with more partners taking paternity leave and childcare responsibilities, future research should investigate subgroups of partners at risk of not adhering to the WHO physical activity guidelines to ensure that comprehensive interventions encompass both parents.

### 4.4 Strengths and limitations

One notable limitation of our study is the exclusion of mothers who gave birth after the age of 40 years. The number of women in this age group is increasing in Western societies (eurostat, 2024). Unfortunately, due to data constraints, we were unable to include this subgroup in our analysis. Future research should aim to include this growing demographic to provide a more comprehensive understanding of physical activity patterns across different age groups of mothers.

The primary outcome measure, weekly hours of MVPA, was based on self-reports (Rosendahl Jensen, 2022). Consequently, there is a risk of social desirability bias and recall bias (Skender et al., 2016). Furthermore, data from the DNHS^33^ was collected in 2021 during the COVID-19 pandemic where politicians authorized limited social interactions and closure of community facilities. This period is likely to have influenced the behaviour of the Danish population, including physical activity patterns (Gjestvang et al., 2022). Consequently, the proportions of mothers not adhering to the WHO physical activity guidelines are notably higher compared with previous years (Jensen et al., 2018). In comparison, a pooled analysis of global physical activity trends in 2001–2016 found that 42 % of women in high-income Western countries did not adhere to the guidelines (Guthold et al., 2018). However, even if the COVID-19 pandemic did influence the reported weekly minutes of MVPA and data of physical activity was biased due to self-report, this would likely concern all mothers, and comparisons between groups would still yield valid results.

The categorization of the exposure, age of youngest child, into five groups based on nursery, kindergarten, and school age in Denmark (Lifeindenmark, 2024; Denmark, 2024b) may hinder comparisons with international studies due to the variations in daycare practices across countries. However, the tendency that mothers of young children are less likely to adhere to WHO physical activity guidelines compared with mothers of older children is observed across countries (Adamo et al., 2012; Candelaria et al., 2012; Gaston et al., 2014; Makama et al., 2023; Kwon et al., 2023; Simpson et al., 2022; Palomäki et al., 2023), which helps to support our findings, despite variations in the categorization of child age.

**Fig. 3:**
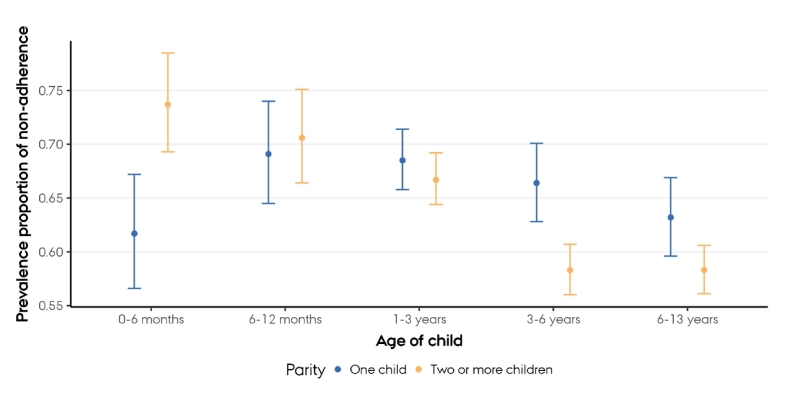
Proportions and 95 % confidence intervals of Danish mothers not adhering to the World Health Organization guidelines of a weekly minimum of 150 min of moderate-to-vigorous physical activity across the five youngest child age groups, stratified by parity.

Thirdly, our stratified analysis on parity revealed a higher prevalence proportion of first-time mothers of children aged 3–6 years not adhering to the WHO physical activity guidelines compared with multipara mothers of children in the same age group (Fig. 3). This finding could be explained, in part, by a significant proportion of these first-time mothers being pregnant when reporting their physical activity in the DNHS 2024.

Despite these limitations, the study is the first of its kind conducted in a Danish population. To the best of our knowledge, the study is the largest population-based study, on a global scale, to investigate physical activity variations in mothers based on the age of their children.

## 5 Conclusion

The proportion of non-adherers to the WHO physical activity guidelines were significantly higher among mothers of infants and toddlers aged 0–3 years as their youngest child compared with mothers of school children aged 6–13 years as their youngest child. Due to the health benefits derived from adequate levels of physical activity, the large proportion of mothers not adhering to the WHO physical activity guidelines is a public health concern. Mothers of infants and toddlers represent a specific subgroup of the population who may need additional support to enhance their physical activity level, thereby contributing to better overall health, which may ultimately benefit both the mothers and their children.

## Declaration of generative AI and AI-assisted technologies in the writing process

During the preparation of this work the authors utilized AI tools such as ChatGPT and Scite to enhance the readability and language of the present paper. After using these tools, the authors reviewed and edited the content as needed and take full responsibility for the content of the publication.

## Funding

This research did not receive any specific grant from funding agencies in the public, commercial, or not-for-profit sectors.

## CRediT authorship contribution statement

**Solvej Videbæk Bueno:** Writing – original draft, Project administration, Formal analysis, Data curation, Conceptualization. **Sebastian Deisting Skejø:** Writing – review & editing, Supervision, Formal analysis, Data curation, Conceptualization. **Rasmus Oestergaard Nielsen:** Writing – review & editing, Supervision, Formal analysis, Data curation, Conceptualization. **Knud Ryom:** Writing – review & editing, Supervision, Conceptualization. **Per Kallestrup:** Writing – review & editing, Supervision, Conceptualization. **Peter Elsborg:** Writing – review & editing, Data curation. **Christina Bjørk Petersen:** Writing – review & editing, Data curation. **Julie Sandell Jacobsen:** Writing – original draft, Supervision, Formal analysis, Data curation, Conceptualization.

## Declaration of competing interest

The authors declare that they have no known competing financial interests or personal relationships that could have appeared to influence the work reported in this paper.

## Data Availability

The authors do not have permission to share data.

## Appendix A Supplementary data

Supplementary material Image 1
