# Supplementary material for: Danish mothers of young children adhere less to international physical activity guidelines compared with mothers of older children
Source: Prev Med Rep. 2025 Jan 11;50:102970. doi: 10.1016/j.pmedr.2025.102970 (PMC11815903; doi:10.1016/j.pmedr.2025.102970)
Supplement: Supplementary file 1 — Supplementary material [file mmc1.docx]

| **Supplementary Table A: Non-adherence to the World Health Organization physical activity guidelines* and age of youngest child stratified by educational level (years) in mothers participating in the Danish National Health Survey 2021** | | | | | | |
| --- | --- | --- | --- | --- | --- | --- |
|  | Living alone | | | Living with someone | | |
| Age of youngest child | n non-adherers /  n total | Prevalence Proportion % (95%-CI) | Prevalence Proportion Ratio (95%-CI) | n non-adherers  / n total | Prevalence Proportion % (95%-CI) | Prevalence Proportion Ratio (95%-CI) |
| 0-6 months | 22/32 | 68.7 (54.4; 86.8) | 1.115 (0.78; 1.60) | 437/643 | 68.0 (64.5; 71.7) | 1.15 (1.05; 1.27) |
| 6-12 months | 19/29 | 65.5 (50.3; 85.3) | 1.06 (0.71; 1.60) | 525/749 | 70.1 (66.9; 73.5) | 1.19 (1.09; 1.30) |
| 1-3 years | 115/155 | 74.2 (67.6; 81.4) | 1.20 (1.02; 1.43) | 1599/2385 | 67.0 (65.2; 69.0) | 1.14 (1.06; 1.22) |
| 3-6 years | 207/320 | 64.7 (59.7; 70.1) | 1.05 (0.90; 1.23) | 1202/2005 | 60.0 (57.8; 62.1) | 1.02 (0.94; 1.10) |
| 6-13 years | 339/550 | 61.6 (57.7; 65.8) | - | 1125/1906 | 59.0 (56.9; 61.3) | - |

*The World Health Organization recommend a weekly minimum of 150 minutes of moderate-to-vigorous physical activity.

| **Supplementary Table B: Non-adherence to the World Health Organization physical activity guidelines* and age of youngest child stratified by educational level (years) in mothers participating in the Danish National Health Survey 2021** | | | | | | | | | | | | |
| --- | --- | --- | --- | --- | --- | --- | --- | --- | --- | --- | --- | --- |
|  | ≤10 years | | | 11-15 years | | | ≥ 16 years | | | Unknown | | |
| **Age of youngest child** | n non-adherers / n total | Prevalence Proportion % (95%-CI) | Prevalence Proportion Ratio  (95%-CI) | n non-adherers / n total | Prevalence Proportion % (95%-CI) | Prevalence Proportion Ratio (95%-CI) | n non-adherers / n total | Prevalence Proportion % (95%-CI) | Prevalence Proportion Ratio (95%-CI) | n non-adherers / n total | Prevalence Proportion % (95%-CI) | Prevalence Proportion Ratio (95%-CI) |
| 0-6 months | 38/60 | 63.3  (52.2; 76.8) | 0.92  (0.65; 1.29) | 160/226 | 70.8  (65.1; 77.0) | 1.14  (0.98; 1.34) | 220/331 | 66.5  (61.6; 71.8) | 1.21  (1.04; 1.41) | 41/58 | 70.7  (59.9; 83.4) | 1.18  (0.81; 1.71) |
| 6-12 months | 34/44 | 77.3  (65.8; 90.7) | 1.12  (0.84; 1.50) | 168/233 | 72.1  (66.6; 78.1) | 1.17  (1.00; 1.35) | 285/426 | 66.9  (62.6; 71.5) | 1.22  (1.06; 1.40) | 57/75 | 76.0  (66.9; 86.3) | 1.27  (0.91; 1.76) |
| 1-3 years | 108/165 | 65.5  (58.6; 73.1) | 0.95  (0.76; 1.19) | 598/834 | 71.7  (68.7; 74.8) | 1.16  (1.05; 1.28) | 884/1362 | 64.9  (62.4; 67.5) | 1.18  (1.06; 1.32) | 124/179 | 69.3  (62.8; 76.4) | 1.16  (0.85; 1.56) |
| 3-6 years | 136/196 | 69.4  (63.2; 76.1) | 1.00  (0.82; 1.23) | 517/837 | 61.8  (58.6; 65.1) | 0.10  (0.89; 1.12) | 671/1160 | 57.8  (55.1; 60.8) | 1.05  (0.93; 1.19) | 85/132 | 64.4  (56.7; 73.1) | 1.07  (0.77; 1.49) |
| 6-13 years | 170/246 | 69.1  (63.6; 75.1) | - | 663/1071 | 61.9  (59.1; 64.9) | - | 571/1039 | 55.0  (52.0; 58.1) | - | 60/100 | 60.0  (51.1; 70.4) | - |

*The World Health Organization recommend a weekly minimum of 150 minutes of moderate-to-vigorous physical activity.

| **Supplementary Table C: Non-adherence to the World Health Organization physical activity guidelines* and age of youngest child stratified by country of origin in mothers participating in the Danish National Health Survey 2021** | | | | | | | | | |
| --- | --- | --- | --- | --- | --- | --- | --- | --- | --- |
|  | Denmark | | | Western countries | | | Non-western countries | | |
| Age of youngest child | n non-adherers / n total | Prevalence Proportion % (95%-CI) | Prevalence Proportion Ratio (95%-CI) | n non-adherers / n total | Prevalence Proportion % (95%-CI) | Prevalence Proportion Ratio (95%-CI) | n non-adherers / n total | Prevalence Proportion % (95%-CI) | Prevalence Proportion Ratio (95%-CI) |
| 0-6 months | 380/572 | 66.4 (62.7; 70.4) | 1.14 (1.02; 1.27) | 28/40 | 70.0 (57.1; 85.7) | 1.23 (0.80; 1.89) | 51/63 | 81.0 (71.8; 91.3) | 1.05 (0.84; 1.32) |
| 6-12 months | 456/667 | 68.4 (64.9; 72.0) | 1.17 (1.06; 1.30) | 28/40 | 70.0 (57.1; 85.7) | 1.23 (0.80; 1.89) | 60/71 | 84.5 (76.5; 93.4) | 1.10 (0.90; 1.34) |
| 1-3 years | 1460/2192 | 66.6 (64.7; 68.6) | 1.14 (1.06; 1.23) | 100/144 | 69.4 (62.3; 77.4) | 1.22 (0.88; 1.71) | 154/204 | 75.5 (69.8; 81.6) | 0.98 (0.82; 1.17) |
| 3-6 years | 1172/1981 | 59.2 (57.0; 61.4) | 1.02 (0.94; 1.10) | 74/122 | 60.7 (52.6; 70.0) | 1.07 (0.74; 1.54) | 163/222 | 73.4 (67.8; 79.5) | 0.95 (0.80; 1.14) |
| 6-13 years | 1273/2185 | 58.3(56.2; 60.4) | - | 50/88 | 56.8 (47.4; 68.2) | - | 141/183 | 77.0 (71.2; 83.4) | - |

*The World Health Organization recommend a weekly minimum of 150 minutes of moderate-to-vigorous physical activity.

| **Supplementary Table D: Non-adherence to the World Health Organization physical activity guidelines* and age of youngest child stratified by urbanization in mothers participating in the Danish National Health Survey 2021** | | | | | | | | | | | | |
| --- | --- | --- | --- | --- | --- | --- | --- | --- | --- | --- | --- | --- |
|  | >=100,000 inhabitants | | | 20,000-100,000 inhabitants | | | 1,000-20,000 inhabitants | | | <1,000 inhabitants | | |
| Age of youngest child | n non-adherers / n total | Prevalence Proportion %  (95%-CI) | Prevalence Proportion Ratio  (95%-CI) | n non-adherers / n total | Prevalence Proportion  %  (95%-CI) | Prevalence Proportion Ratio  (95%-CI) | n non-adherers / n total | Prevalence Proportion %  (95%-CI) | Prevalence Proportion Ratio  (95%-CI) | n non-adherers / n total | Prevalence Proportion %  (95%-CI) | Prevalence Proportion Ratio  (95%-CI) |
| 0-6 months | 163/240 | 67.9  (62.3; 74.1) | 1.14  (0.95; 1.36) | 70/116 | 60.3  (52.1; 69.9) | 1.03  (0.79; 1.35) | 137/193 | 71.0  (64.9; 77.7) | 1.23  (1.04; 1.46) | 89/126 | 70.6  (63.1; 79.1) | 1.11  (0.90; 1.37) |
| 6-12 months | 178/265 | 67.2  (61.7; 73.1) | 1.13  (0.95; 1.34) | 87/125 | 69.6  (62.0; 78.1) | 1.19  (0.95; 1.50) | 164/232 | 70.7  (65.1; 76.8) | 1.23  (1.04; 1.44) | 115/156 | 73.7  (67.1; 81.0) | 1.16  (0.96; 1.34) |
| 1-3 years | 529/824 | 64.2  (61.0; 67.6) | 1.08  (0.94; 1.23) | 291/452 | 64.4  (60.1; 69.0) | 1.10  (0.93; 1.30) | 559/794 | 70.4  (67.3; 73.7) | 1.22  (1.09; 1.37) | 335/470 | 71.3  (67.3; 75.5) | 1.12  (0.97; 1.29) |
| 3-6 years | 375/634 | 59.1  (55.4; 63.1) | 0.99  (0.85; 1.15) | 265/441 | 60.1  (55.7; 64.8) | 1.03  (0.86; 1.23) | 481/799 | 60.2  (56.9; 63.7) | 1.04  (0.92; 1.18) | 288/451 | 63.9  (59.6; 68.4) | 1.0  (0.86; 1.17) |
| 6-13 years | 339/568 | 59.7  (55.8; 63.9) | - | 251/429 | 58.5  (54.0; 63.4) | - | 538/932 | 57.7  (54.6; 61.0) | - | 336/527 | 63.8  (59.8; 68.0) | - |

*The World Health Organization recommend a weekly minimum of 150 minutes of moderate-to-vigorous physical activity.

| **Supplementary Table E: Non-adherence to the World Health Organization physical activity guidelines* and age of youngest child stratified by family income (tertiles) in mothers participating in the Danish National Health Survey 2021** | | | | | | | | | |
| --- | --- | --- | --- | --- | --- | --- | --- | --- | --- |
|  | 1st | | | 2nd | | | 3rd | | |
| Age of youngest child | n non-adherers / n total | Prevalence Proportion % (95%-CI) | Prevalence Proportion Ratio (95%-CI) | n non-adherers / n total | Prevalence Proportion % (95%-CI) | Prevalence Proportion Ratio (95%-CI) | n non-adherers / n total | Prevalence Proportion % (95%-CI) | Prevalence Proportion Ratio (95%-CI) |
| 0-6 months | 177/240 | 73.8 (68.4; 79.5) | 1.12 (0.97; 1.29) | 128/198 | 64.6 (58.3; 71.7) | 1.06 (0.88; 1.28) | 154/237 | 65.0 (59.2; 71.3) | 1.26 (1.05; 1.50) |
| 6-12 months | 180/240 | 75.0 (69.7; 80.7) | 1.14 (0.99; 1.31) | 183/268 | 68.3 (62.9; 74.1) | 1.12 (0.96; 1.31) | 181/270 | 67.0 (61.7; 72.9) | 1.30 (1.10; 1.53) |
| 1-3 years | 569/798 | 71.3 (68.2; 74.5) | 1.08 (0.98; 1.20) | 623/889 | 70.1 (67.1; 73.2) | 1.15 (1.03; 1.28) | 522/853 | 61.2 (58.0; 64.6) | 1.18 (1.04; 1.35) |
| 3-6 years | 485/746 | 65.0 (61.7; 68.5) | 1.00 (0.88; 1.10) | 466/771 | 60.4 (57.1; 64.0) | 0.99 (0.88; 1.12) | 458/808 | 56.7 (53.4; 60.2) | 1.10 (0.95; 1.26) |
| 6-13 years | 555/842 | 65.9 (62.8; 69.2) | - | 490/804 | 60.9 (57.7; 64.4) | - | 419/810 | 51.7 (48.4; 55.3) | - |

*The World Health Organization recommend a weekly minimum of 150 minutes of moderate-to-vigorous physical activity.
